# High-Definition DNA Methylation Profiles from Breast and Ovarian Carcinoma Cell Lines with Differing Doxorubicin Resistance

**DOI:** 10.1371/journal.pone.0011002

**Published:** 2010-06-08

**Authors:** Michael Boettcher, Frank Kischkel, Jörg D. Hoheisel

**Affiliations:** 1 Division of Functional Genome Analysis, Deutsches Krebsforschungszentrum, Heidelberg, Germany; 2 TherapySelect GmbH & Co. KG, Heidelberg, Germany; Duke University, United States of America

## Abstract

Acquired drug resistance represents a frequent obstacle which hampers efficient chemotherapy of cancers. The contribution of aberrant DNA methylation to the development of drug resistant tumor cells has gained increasing attention over the past decades. Hence, the objective of the presented study was to characterize DNA methylation changes which arise from treatment of tumor cells with the chemotherapeutic drug doxorubicin. DNA methylation levels from CpG islands (CGIs) linked to twenty-eight genes, whose expression levels had previously been shown to contribute to resistance against DNA double strand break inducing drugs or tumor progression in different cancer types were analyzed. High-definition DNA methylation profiles which consisted of methylation levels from 800 CpG sites mapping to CGIs around the transcription start sites of the selected genes were determined. In order to investigate the influence of CGI methylation on the expression of associated genes, their mRNA levels were investigated via qRT-PCR. It was shown that the employed method is suitable for providing highly accurate methylation profiles, comparable to those obtained via clone sequencing, the gold standard for high-definition DNA methylation studies. In breast carcinoma cells with acquired resistance against the double strand break inducing drug doxorubicin, changes in methylation of specific cytosines from CGIs linked to thirteen genes were detected. Moreover, similarities between methylation profiles obtained from breast and ovarian carcinoma cell lines with acquired doxorubicin resistance were found. The expression levels of a subset of analyzed genes were shown to be linked to the methylation levels of the analyzed CGIs. Our results provide detailed DNA methylation information from two separate model systems for acquired doxorubicin resistance and suggest the occurrence of similar methylation changes in both systems upon exposure to the drug.

## Introduction

This study was designed to investigate epigenetic alterations which arise from treatment of tumor cells with the anthracycline antibiotic, doxorubicin [1]. Early stage and metastatic breast cancer, as well as platinum-refractory/-resistant ovarian cancer is commonly treated by means of liposomal doxorubicin either as monotherapy or in combination with other chemotherapeutic drugs [2], [3]. Acquired resistance, however, frequently prevents successful doxorubicin treatment of those diseases. Epigenetic alterations are potential driving forces for acquired chemoresistance [4]. A typical epigenetic modification, which is frequently observed in tumor cells, is aberrant methylation of cytosine bases (C) located 5′ of a guanine base (G), so called CpG dinucleotides [5]. Although CpG dinucleotides are generally underrepresented in mammalian genomes, they frequently cluster around the transcription start site (TSS) of genes, in genomic areas referred to as CpG islands (CGIs) [6]. Genome-wide hypo-methylation, in combination with CGI specific hyper-methylation, is a common hallmark of cancer development [7]. Hyper-methylation of CGIs located in the promoter region of a variety of genes implicated in cell cycle, invasion, apoptosis, DNA repair and drug transport has been linked to transcriptional silencing of the associated genes [4], [8]. Probably the most prominent gene involved in drug resistance being transcriptionally regulated via CGI methylation is *ABCB1* (*MDR1*), encoding the drug efflux transporter P-glycoprotein. A number of studies have shown that over-expression of *ABCB1* can render cell lines resistant to a wide range of chemotherapeutic drugs, including doxorubicin [9], [10]. However, not only drug transporters but also, genes involved in metabolizing drugs, repairing the cellular damage caused by them as well as inducing apoptosis in cells that have been irreparably damaged play a crucial role in the development of drug resistance, [11], [12].

Based on these considerations, twenty-eight genes whose levels of expression were previously linked to resistance of different cancer types to DNA double strand break (DSB) inducing drugs were selected from literature. Furthermore, each of those genes was selected to contain one or more CGIs close to its TSS. By means of microarray hybridization, high-definition methylation profiles were recorded covering thirty-three CGIs associated with the twenty-eight selected genes. We used this method to determine methylation profiles from five carcinoma cell lines representing two cancer types commonly treated via the DSB inducing chemotherapeutic drug doxorubicin.

In order to study the effects of acquired doxorubicin resistance on DNA methylation in breast cancer, we examined methylation levels in the cell lines MCF-7_wt and MCF-7_ADR. Seeing as MCF-7_ADR was a doxorubicin selected sub-line of MCF-7_wt, both cell lines originally exhibited identical genetic as well as epigenetic backgrounds. Acquired changes in the epigenome of MCF-7_ADR thus were attributable to the effects of doxorubicin selection. We further examined CGI methylation levels in the ovarian carcinoma cell lines OVCAR-4, OVCAR-5 and NCI/ADR-RES. Unlike MCF-7_wt/_ADR, each of those three cell lines originated from different patients and therefore exhibited dissimilar genetic and epigenetic backgrounds. Moreover, OVCAR-4 and OVCAR-5 both represented non-doxorubicin selected cell lines, hence differences in their doxorubicin tolerance derived from inherent resistance. NCI/ADR-RES, on the other hand, constituted a doxorubicin selected sub-line originating from the ovarian carcinoma cell line OVCAR-8 [13] and served as a model for acquired doxorubicin resistance in ovarian carcinoma.

In detail, the questions addressed by this study were (1) how does doxorubicin treatment alter DNA methylation in relevant CGIs *in-vitro*, (2) how do these changes compare between different carcinoma cell lines and (3) are detected methylation changes linked to altered gene expression.

## Results

### Doxorubicin tolerance of investigated cell lines

**Figure 1:**
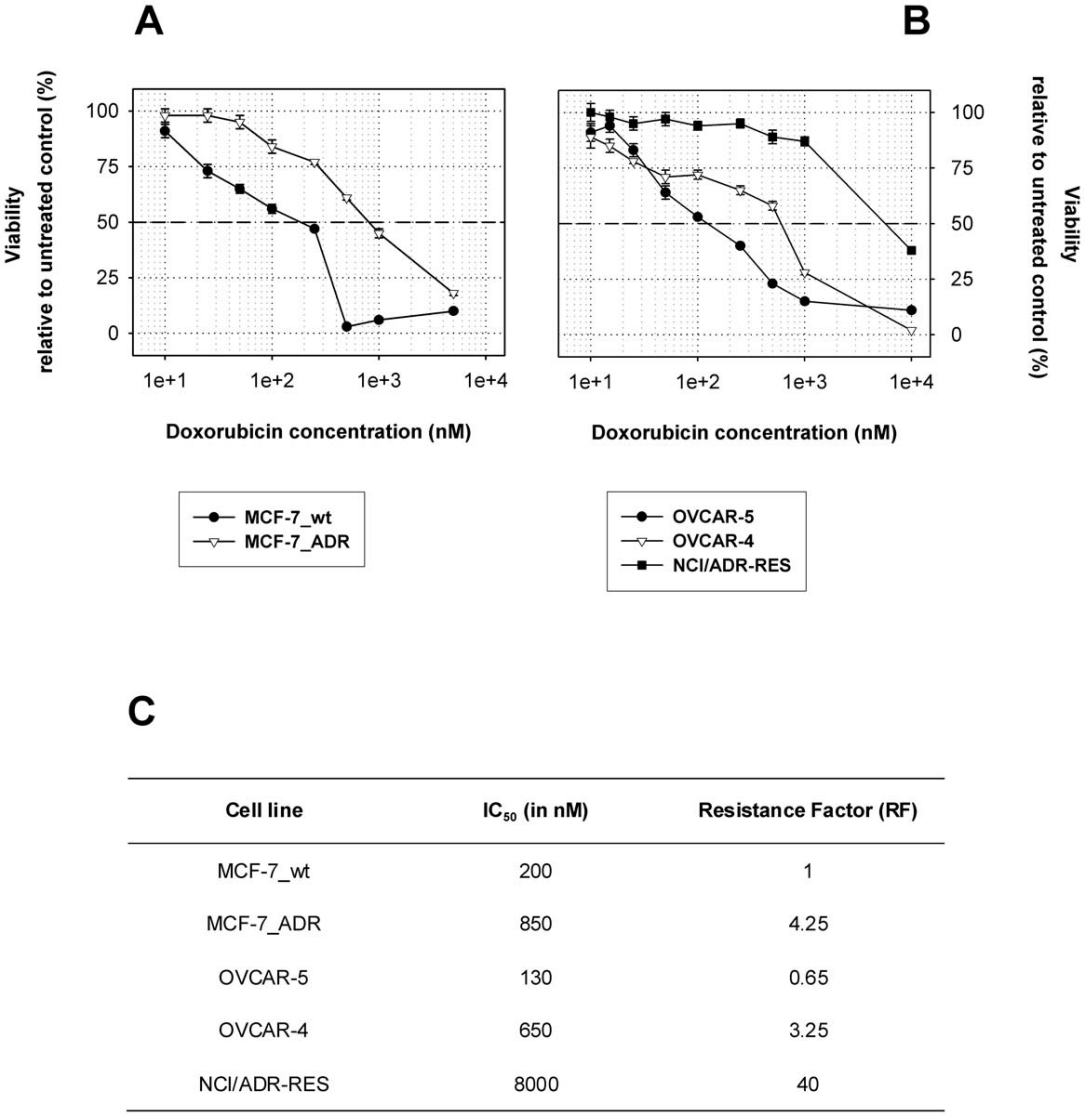
Cell viability after 72 hours of doxorubicin treatment. **A**: Viability assay of MCF-7_wt and MCF-7_ADR. Dashed line indicates 50 percent viability of untreated control. **B**: Viability assay of OVCAR-5, OVCAR-4 and NCI/ADR-RES. **C**: Summary of the determined IC_50_ values from each cell line as well as resistance relative to MCF-7_wt (RF).

For each carcinoma cell line investigated in this study, viability assays were performed in order to determine their tolerance against doxorubicin (Figure 1). As expected, IC_50_ values differed significantly and documented the cells resistance status.

### Detection of CpG methylation levels

**Figure 2:**
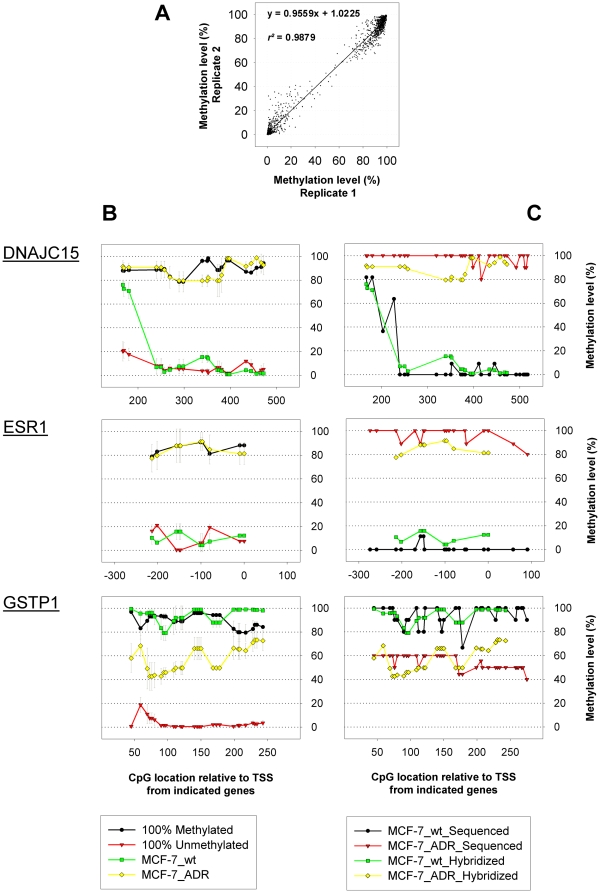
Methylation profiling – Reproducibility and validation. **A**: Reproducibility, illustrated via two replicate hybridizations, of fully methylated and fully unmethylated control fragment pools. **B**: Methylation profiles determined from CGIs around the TSS of the genes *DNAJC15*, *ESR1* and *GSTP1*. Shown are hybridizations of fully methylated and unmethylated control fragments as well as fragments amplified from the cell lines MCF-7_wt and MCF-7_ADR. **C**: Validation of methylation profiles via clone sequencing of the PCR fragments used for microarray hybridizations shown in Figure 2B.

We employed microarray technology for the detection of methylation levels from CpG sites. For that purpose, CGIs of interest were PCR amplified from sodium bisulfite converted genomic DNA (gDNA) of each cell line, changing unmethylated CpG dinucleotides into TpG while leaving methylated ones unchanged [14]. The exact primer sequences and annealing temperatures used for PCR amplification are shown in Table S1. From each sample, pools of labeled PCR-fragments were hybridized to microarrays containing 25 nucleotide long probe sequences representing the formerly unmethylated TpG- as well as methylated CpG-containing sequences. The ratio (CpG/(CpG+TpG))×100 calculated from both probe signal intensities provided a measure of the methylation level for each CpG site in percent [15]. In order to determine the potential of each probe sequence to detect different levels of methylation from the selected CpG sites, we hybridized control pools of *in-vitro* methylated as well as unmethylated PCR fragments to individual microarrays. Only probe sequences exhibiting a methylation ratio above 75% for the fully methylated control, together with less than 25% for the unmethylated control were considered for subsequent analysis. The results from two replicates of independently labeled and hybridized control pools presented in Figure 2A illustrate the high reproducibility of the method (*r^2^* = 0.9879). Figure 2B further gives an overview of the methylation profiles obtained from three CGIs associated with the genes *DNAJC15*, *ESR1* and *GSTP1* respectively. Cytosine methylation levels from CpG sites within the three different CGIs presented in Figure 2B were additionally quantified via sequencing of nine or more sub-cloned PCR products from the two cell lines MCF-7_wt and MCF-7_ADR. In total, the methylation levels from 130 CpG sites were determined by means of microarray hybridization as well as sequencing and revealed a strong correlation with a coefficient of *r^2^* = 0.9258, indicating the high accuracy of the described method. Methylation profiles detected via hybridization compared to sequencing analysis are shown in Figure 2C.

### CGI methylation changes linked to acquired doxorubicin resistance in breast carcinoma cells

We identified CGIs associated with thirteen genes to display changes in methylation levels between MCF-7_wt and MCF-7_ADR. Table 1 summarizes the tendencies of methylation changes between both breast carcinoma cell lines as well as the three investigated ovarian carcinoma cell lines, with plus (+) indicating hyper-methylation with acquired doxorubicin resistance and minus (−) indicating hypo-methylation. CGIs associated with the genes *ABCG2*, *APAF1*, *ARHGEF2*, *AVEN*, *BAD*, *BIRC5*, *CDKN2A*, *FANCF*, *FOXO3A*, *MLH1*, *MSH2*, *PTEN* and *RALBP1* did not show detectable levels of methylation in any cell line and hence are not listed in Table 1. Table S2 summarizes the determined methylation levels from all analyzed CpG sites in each of the five cell lines.

**Table 1:** Tendency of methylation level alterations linked to doxorubicin resistance.

	Breast carcinoma	Ovarian carcinoma
*ABCB1*	−	−
*APC*	−	−
*BRCA1*	+	+
*CDH1*	+	+
*DNAJC15*	+	+
*ESR1*	+	none
*GSTP1*	−	none
*HIC1*	−	−
*IGFBP3*	none	−
*PLAU*	−	none
*RAB6C*	+	none
*RASSF1*	−	none
*SULF2*	+	+
*TGM2*	−	none

Hypo-methylation of doxorubicin resistant compared to sensitive cell lines is indicated by “minus” (−), while hyper-methylation is indicated by “plus” (+).Cells marked with “none” represent CGIs of consistently high or low levels of methylation between the analyzed cell lines.

### Overlapping tendencies between breast and ovarian carcinoma cell lines

When comparing the alterations in methylation levels between the cell lines MCF-7_wt/_ADR to changes observed in the ovarian carcinoma cell lines, we found seven CGIs to display the same tendencies. Specifically, we identified hyper-methylation in CGIs associated with *BRCA1*, *CDH1*, *DNAJC15* and *SULF2* as well as hypo-methylation for *ABCB1*, *APC* and *HIC1* with increased doxorubicin tolerance (Table 1). Interestingly, the same tendencies were observed in OVCAR-4 (inherent resistance) as well as in NCI/ADR-RES (acquired resistance) when compared to OVCAR-5. Additionally, we detected hypo-methylation in a CGI linked to *IGFBP3* with increased doxorubicin tolerance, which was not observed in breast carcinoma cell lines.

### Gene expression levels of CGI associated genes

In order to determine the impact of CGI methylation on the expression of associated genes, we detected mRNA levels of a subset of genes via qRT-PCR. The normalized results relative to expression levels in MCF-7_wt are summarized in Table S3.

In the case of the breast carcinoma cell lines MCF-7_wt/_ADR, the methylation status of CGIs associated with the genes *ABCB1*, *BRCA1*, *CDH1*, *DNAJC15*, *ESR1*, *GSTP1*, *PLAU*, *SULF2* and *TGM2* was connected to the expression of the genes. This was assumed to be the case for concomitant mRNA up-regulation after CGI hypo-methylation as well as mRNA down-regulation after CGI hyper-methylation. In case of the ovarian carcinoma cell lines OVCAR-5, OVCAR-4 and NCI/ADR-RES, the methylation status of CGIs associated with the genes *ABCB1*, *BRCA1*, *CDH1*, *DNAJC15*, and *SULF2* was connected to the expression of the genes.

In addition to genes linked to differentially methylated CGIs, we determined mRNA levels of the gene *ABCG2*, encoding a putative doxorubicin efflux transporter [16]. The cell lines most sensitive to doxorubicin (MCF-7_wt, OVCAR-5) expressed much higher levels of *ABCG2* than cell lines more resistant to the drug (MCF-7_ADR, OVCAR-4, NCI/ADR-RES). Consequently, *ABCG2* expression is not likely to play a significant role in the mechanism causing resistance in the examined cell lines. We further determined *TOP2A* transcript levels; a primary target of doxorubicin [17], [18], and found slightly decreased mRNA levels in doxorubicin resistant cell lines, which might contribute to their resistance (Table S3).

## Discussion

Comparison of CGI methylation profiles from the breast carcinoma cell line MCF-7_wt with profiles from its doxorubicin selected counterpart MCF-7_ADR, allowed us to attribute changes in methylation levels at specific CpG sites to acquired doxorubicin resistance. Between both cell lines, we found aberrant CGI methylation profiles linked to thirteen out of twenty-eight genes. The identified genes are involved in drug transport and detoxification (*ABCB1* [9], [19], [20], [21], [22], *DNAJC15* [23], [24], *GSTP1* [19], [21], [22], *RAB6C* [25], [26]), DNA damage repair (*BRCA1* [27], [28]) as well as tumor cell proliferation/invasion (*APC* [29], [30], [31], *CDH1* [29], [32], *ESR1* [12], [33],[34], *HIC* [29], *PLAU* [22], [35], *RASSF1* [29], [30], [31], *SULF2* [36], [37], *TGM2* [12], [38]). An overlapping set of seven genes (*ABCB1*, *APC*, *BRCA1*, *CDH1*, *DNAJC15*, *HIC1* and *SULF2*) displayed the same methylation changes in the examined set of ovarian carcinoma cell lines. An overview of methylation tendencies with acquired doxorubicin resistance is given in Table 1. For clarity reasons, not all of the identified alterations are explicitly discussed in the following sections, but rather a selection of those which illustrate the most important findings of this study. The full data sets from each analyzed cell line, consisting of methylation levels from 800 CpG sites as well as the mRNA levels from a subset of analyzed genes are summarized in Tables S2 and S3 respectively.

### Hyper- and hypo-methylation events occur in distinct CGI sub-regions with increased doxorubicin resistance

**Figure 3:**
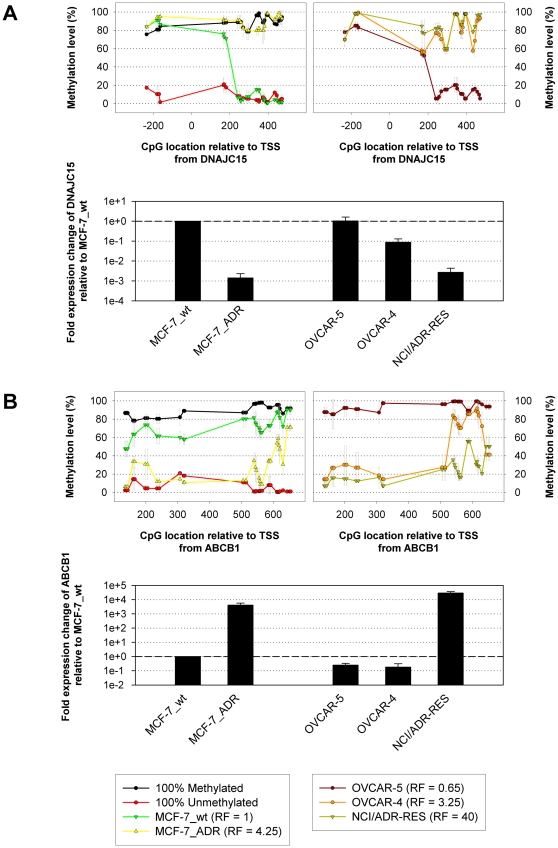
Hyper- and hypo-methylation events occur in distinct CGI sub-regions with increased doxorubicin resistance. **A**: A sub-region of a CGI associated with the gene *DNAJC15* becomes hyper-methylated and the gene's mRNA levels decrease with increased resistance. **B**: A sub-region of a CGI associated with the gene *ABCB1* becomes hypo-methylated and the gene's mRNA levels increase with increased resistance. Methylation profiles from CGIs linked to all analyzed twenty-eight genes are shown in Table S2 and expression levels of a subset of those genes in Table S3.

An important issue when analyzing DNA methylation levels is the heterogeneity displayed by many CGIs [39]. While some of the analyzed islands exhibited almost identical levels of methylation at each CpG site, some others showed considerable variation in methylation levels between distinct CGI sub-regions, as was the case for *DNAJC15* and *ABCB1* (Figure 3). For that reason, it is essential to study changes in CGI methylation by means of a method that allows high-definition analysis. Methylation profiles from CGIs associated with *DNAJC15*, a putative inhibitor of *ABCB1* transcription [40], displayed high levels of methylation between 200 nt upstream and 200 nt downstream from the TSS in all analyzed cell lines (Figure 3). In the region between 200 nt and 400 nt downstream from the TSS, however, methylation levels differed dramatically between sensitive cell lines and cell lines with acquired doxorubicin resistance. In MCF-7_wt as well as in OVCAR-5, methylation in the latter region was almost absent and *DNAJC15* mRNA levels were high (Figure 3). In the more resistant cell lines, the complete CGI was found to be hyper-methylated and, accordingly, mRNA levels were significantly lower. These findings were consistent with results from Strathdee *et al.* [23], who identified methylation of the same CGI within the first exon of *DNAJC15* to be responsible for the gene's transcriptional regulation. Moreover, they linked loss of *DNAJC15* expression to resistance to the chemotherapeutic drug cisplatin in an ovarian carcinoma cell line as well as in ovarian carcinoma patients [24]. Here we provided additional evidence for the epigenetic regulation of *DNAJC15* expression in breast and ovarian carcinoma cell lines and further demonstrated the correlation of the gene's loss-of-expression with resistance to doxorubicin.

Moreover, consistent with the proposed inhibitory function of *DNAJC15* on the expression of *ABCB1* [40], we found increased levels of *ABCB1* mRNA in cell lines with decreased *DNAJC15* levels (Figure 3). One exception, however, was the inherently resistant cell line OVCAR-4, which exhibited 10-fold decreased *DNAJC15* mRNA levels compared to OVCAR-5, but almost identical levels of *ABCB1*. This finding might be explained by epigenetic silencing of *ABCB1* in OVCAR-4, preventing expression despite reduced levels of its inhibitor *DNAJC15*. One possible explanation of the gene's transcriptional silencing in OVCAR-4 was given by its CGI methylation profile shown in Figure 3. While CpG sites upstream of 500 nt from the TSS showed no methylation, downstream CpG sites were highly methylated. Hence the identified region is of potential relevance for the epigenetic silencing of *ABCB1* expression.

We further identified CGIs linked to the genes *ESR1*, *HIC1*, *IGFBP3*, *SULF2*, *TGM2* and *TP73* to exhibit distinct methylation sub-regions similar to the ones observed in *DNAJC15* and *ABCB1* (Table S2). Taken together, these findings highlight the importance of high-definition profiling for the precise mapping of drug resistance associated changes of DNA methylation.

### CGI associated with *GSTP1* becomes partially hypo-methylated with acquired doxorubicin resistance

The enzyme, glutathione S-transferase P1, encoded by the gene *GSTP1*, has long been known to conjugate drugs, including doxorubicin, with glutathione, resulting in their detoxification [41]. Its expression has previously been linked to doxorubicin resistance in ovarian carcinoma cell lines and patients [42], [43]. We found the CGI located within the gene's first exon to display continuously high levels of methylation in the doxorubicin sensitive cell line MCF-7_wt, decreasing to about 50 percent in MCF-7_ADR (Figure 2B). These findings were confirmed via clone sequencing (Figure 2C) and thus further illustrate the presented method's potential to quantify levels of DNA methylation.

**Figure 4:**
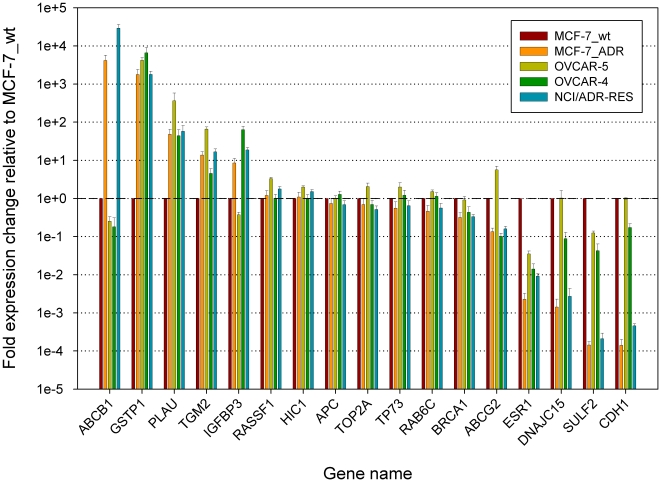
Expression levels from a subset of genes. Levels of mRNA from indicated genes in the cell lines MCF-7_wt, MCF-7_ADR, OVCAR-5, OVCAR-4 and NCI/ADR-RES in logarithmic scale relative to MCF-7_wt. Error bars indicate standard deviation between triplicates. Values are summarized in Table S3.

Seeing as the cell line MCF-7_ADR derived from one single clone, the existence of two sub-populations, each carrying exclusively methylated or unmethylated copies of *GSTP1* associated CGIs, is unlikely. A more plausible explanation would be hypo-methylation of the CGI associated with one of two copies of *GSTP1* during the doxorubicin selection process, while the second one remained methylated. Concomitantly, *GSTP1* mRNA levels in MCF-7_ADR were strongly increased when compared to MCF-7_wt, similar to the levels observed in each of the three completely unmethylated ovarian carcinoma cell lines (Figure 4). These findings suggest DNA methylation in the CGI of the gene's first exon to be involved in the transcriptional regulation of *GSTP1*. While *GSTP1* mRNA levels are highly different between MCF-7_ADR and MCF-7_wt; they are largely the same between the analyzed ovarian carcinoma cell lines. These results argue against *GSTP1* expression levels being a major determinant of doxorubicin tolerance in OVCAR-5, OVCAR-4 and NCI/ADR-RES.

### Acquired doxorubicin resistance decreased methylation of hyper-methylated tumor markers

CGIs associated with three putative tumor suppressor genes *APC*, *HIC1* and *RASSF1* have previously been shown to be frequently hyper-methylated in breast and ovarian tumors when compared to healthy tissue [29], [30], [31]. Furthermore, promoter hyper-methylation of *APC* and *RASSF1* has been demonstrated to be a significant prognostic factor for the survival of breast cancer patients [30]. Accordingly, we detected high levels of CGI methylation for *APC*, *HIC1* and *RASSF1* in the breast carcinoma cell line MCF-7_wt and for *APC* and *HIC1* in the doxorubicin sensitive cell line OVCAR-5. Surprisingly, in carcinoma cell lines of acquired doxorubicin resistance (MCF-7_ADR, NCI/ADR-RES), methylation of CGIs from *APC*, *HIC1* and *RASSF1* was strongly reduced or completely absent (Table S2). Interestingly, none of the observed changes in CGI methylation levels were linked to changes in mRNA levels of the associated genes (Figure 4). These findings suggest that hypo-methylation of certain CGIs that typically become hyper-methylated during carcinogenesis might be a common event in breast and ovarian carcinoma cell lines with acquired doxorubicin resistance, but does not lead to re-expression of associated genes.

### Interplay between DNA methylation and chromatin remodeling

In addition to genes such as *APC*, *HIC1* and *RASSF1* which show altered DNA methylation, but no concomitant change in gene expression levels we found a number of genes to display altered gene expression levels but no concomitant change in DNA methylation. This issue is best illustrated by means of the examined CGI associated with the gene *ABCG2*. While no DNA methylation could be detected in the analyzed region in any of the cell lines (Table S2), gene expression levels differed significantly between them (Figure 4). In this respect it is important to keep in mind that DNA methylation changes on their own do not regulate gene expression levels, but that this process is tightly coupled to chromatin modifications such as histone acetylation, methylation or phosphorylation [44], [45]. There is evidence that histone modification and subsequently altered gene expression can precede DNA methylation changes [5], [46]. Hence, in the example of *ABCG2* it is possible that histone modifications influence its expression independently from DNA methylation in the examined region.

### Breast and ovarian carcinoma cell lines with acquired doxorubicin resistance display similar methylation profiles

When we compared methylation levels from all 800 CpG sites between all breast and ovarian carcinoma cell lines analyzed in this study, we found methylation profiles from the breast carcinoma cell line MCF-7_ADR and the ovarian carcinoma cell line NCI/ADR-RES to be the most similar (Table 2). Given that both cell lines originated from different cancer types but were selected for doxorubicin resistance, this finding strongly suggested similar changes in methylation patterns during formation of resistance in breast and ovarian carcinoma cells. In comparison, we found no correlation between profiles from the breast carcinoma cell line MCF-7_ADR and its parental counterpart MCF-7_wt (Table 2), further illustrating the profound alterations of methylation patterns during the acquisition of doxorubicin resistance.

**Table 2:** Correlation coefficients (*r^2^*) display strongest similarity between methylation profiles from cell lines with acquired doxorubicin resistance.

MCF-7_wt	OVCAR-5	OVCAR-4	MCF-7_ADR	NCI/ADR-RES	Cell line
	0.0409	0.0239	0.0551	0.0001	**MCF-7_wt**
		0.2859	0.1439	0.1465	**OVCAR-5**
			0.3776	0.5330	**OVCAR-4**
				0.6311	**MCF-7_ADR**
					**NCI/ADR-RES**

Shown are correlation coefficients (*r^2^*) between methylation profiles from indicated cell lines. Each methylation profile consists of methylation levels from 800 CpG sites.

### Conclusions

The detected profiles represent highly accurate comprehensive pictures of CGI methylation from sets of selected putative drug resistance genes. Pre-selection of genes allowed the detailed analysis of methylation profiles from genes of potential relevance to doxorubicin resistance. It was found that several CGIs exhibited doxorubicin-related hyper- as well as hypo-methylation only at specific CpG positions. These findings illustrate the importance of high-definition profiling as compared to the analysis of only individual CpG sites.

## Materials and Methods

### Selection of MCF-7_ADR cells from MCF-7_wt

MCF-7_wt cells were exposed to increasing concentrations of doxorubicin, initially to 2-fold the IC_50_ for 24 hours, followed by washing and incubation in drug-free culture medium until new colonies had formed. This procedure was repeated several times, each time doubling the original IC_50_ up until 64-fold the original IC_50_ was reached. Surviving cells were subjected to a doxorubicin dilution series ranging from 16- to 512-fold the original IC_50_. Cells which proliferated at the highest drug concentration within one week were considered chemotherapy refractory. Resistant colonies were picked from cells treated with 128-fold the original IC_50_ and expanded in continuous presence of 10-fold the original IC_50_. The cell line MCF-7_wt and the doxorubicin selected subline MCF-7_ADR was kindly supplied by the laboratory of Dr. Ralf A. Hilger. Before the final cell viability assays, cells were cultured in doxorubicin-free medium for two weeks.

### Cell culture conditions and viability assays

MCF-7_wt and MCF-7_ADR cells were cultured in DMEM (10% FCS, 1% penicillin/streptomycin 10,000 U) and OVCAR-5, OVCAR-4 and NCI/ADR-RES cells were obtained directly from the National Cancer Institute and cultured in RPMI (10% FCS, 1% penicillin/streptomycin 10,000 U) under standard cell culture conditions (37°C, 5% CO_2_). For viability assays, cells were seeded in triplicate into 96 well microplates at 1,000 as well as 2,000 cells per well. Twenty-four hours later, cells were treated with different concentrations of doxorubicin. Seventy-two hours post treatment, cells were incubated with 50 µl medium containing resazurine (20 µg/ml) and incubated for one to four hours before detection of fluorescence (Ex: 544 nm/Em: 590 nm). After background subtraction, cell viability from every drug concentration was normalized to the untreated control.

### Amplification and labeling of sample targets

In order to analyze methylation levels from the cell lines of interest, total genomic DNA (gDNA) was isolated from each cell line using the QIAamp DNA Micro Kit (Qiagen, Hilden, Germany) according to the manufacturer's instructions. Following purification, 2 µg of gDNA from each cell line were subjected to sodium bisulfite treatment via the EpiTect Bisulfite Kit (Qiagen) according to the manufacturer's instructions. Each CGI of interest was PCR amplified using the primer pair given in Table S1. Primer pairs were designed using the web-based software MethPrimer [47] and their optimal annealing temperature was determined via gradient PCR. The exact sizes of each PCR product (ranging from 198 nt up to 777 nt in size) as well as Ensembl transcript IDs and exon IDs from the examined sequences are summarized in Table S1. Genomic regions of interest were amplified by means of PCR using 20 ng of purified sodium bisulfite treated template DNA, 0.4 µM forward and 0.4 µM reverse primer, 250 µM of each dNTP (Fermentas, St. Leon-Rot, Germany), 1× HotStart Buffer (Qiagen), 1× Q-Solution (Qiagen), 1.5 mM MgCl_2_, 2.5 units HotStart polymerase (Qiagen) and in a total volume of 50 µl. Thermal cycler PCR conditions were 95°C for 15 min followed by seven cycles of 95°C for 1:00 min, 64°C for 2:00 min, 72°C for 2:00 min with a decreased annealing temperature of 1°C in each cycle before an additional 39 cycles of 95°C for 0:40 min, optimized annealing temperature (see Table S1) for 2:00 min, 72°C for 1:30 min and finally 72°C for 10:00 min. PCR products were purified using Millipore MultiScreen PCR_μ96_ filter plates according to the manufacturer's instructions. For every individual cell line of interest, equimolar amounts of all purified PCR fragments were pooled. For labeling, 300 ng from each of those PCR product pools were incubated together with 30 ng/µl random primer oligonucleotides (Invitrogen, Karlsruhe, Germany) in a total volume of 28 µl at 99°C for 5 min. After the denaturation step 1× reaction buffer (1 M Hepes pH 6.6, 250 mM Tris-HCl pH 8.0, 25 mM MgCl_2_, 50 mM 2-mercaptoethanol), 2 mM of each dATP, dCTP, dGTP and 1.3 mM dTTP, (Fermentas) together with 0.7 mM biotinylated-dUTP (Roche, Mannheim, Germany), 0.4 mg/ml BSA (Sigma, Hamburg, Germany) and 7.5 units Klenow fragment (New England Biolabs, Frankfurt, Germany) was added to a total volume of 40 µl. After incubation at 37°C for 3 h and 75°C for 10 min, 4 µl of 3 M sodium acetate (pH 5.6) and 100 µl ethanol were added and the DNA was precipitated at −80°C for 2 h. After centrifugation at 18,320×g for 20 min the supernatant was aspirated, the pellet was dried and resuspended in 15 µl 1× hybridization mix (100 mM 2-[N-morpholino]ethanesulfonic acid (MES), 0.9 M NaCl, 20 mM Na_2_EDTA, 0.01% (v/v) Tween-20, 0.5% BSA 0.1 mg/ml herring sperm DNA (Febit Biomed, Heidelberg, Germany).

### Preparation of control targets

From total human gDNA (Invitrogen), fragments containing each CGI of interest, including the primer binding sites shown in Table S1, were amplified via PCR. From each fragment, 1 µg was incubated at 37°C for 3 h together with 8 units of SssI methyltransferase (NEB, Frankfurt, Germany). To confirm complete in-vitro methylation 200 ng DNA were digested, using 20 units of the methylation sensitive restriction enzyme *Bst*UI (NEB) for two hours at 60°C. From each fully methylated as well as unmethylated target, 1 ng was used for subsequent sodium bisulfite treatment via the EpiTect Bisulfite Kit (Qiagen) followed by a PCR amplification step of each CGI. After purification via QIAquick PCR purification columns (Qiagen), an equimolar pool of fully methylated as well as one of unmethylated control targets were prepared. Both pools were labeled and hybridized the same way as sample target pools.

### Microarray analysis

The photo-controlled *in-situ* synthesis technology Geniom One (Febit Biomed) was used for synthesis, hybridization and detection of microarrays [48]. The Geniom One microarray is divided into eight individually accessible subarrays allowing the analysis of eight samples in parallel. Probe sequences the size of 25 nucleotides were synthesized, resembling the sodium bisulfite converted sequence of each CGI of interest. Every probe sequence was designed to feature at its central position a particular CpG site of interest either as a CpG or a TpG dinucleotide, hence being complementary to the methylated (M) or unmethylated (U) cytosine after sodium bisulfite treatment. Consequently, the microarray layout included 1600 different probe sequences in four replicates. Before hybridization the biotinylated target pools in 1× hybridization mix described above, were heated to 95°C for 3 min then placed on ice for 1 min. The denatured targets were then applied to individual subarrays of the Geniom One microarray and incubated at 45°C for 16 h. After washing routines according to the Febit protocol, each subarray was incubated with 5 µg/ml streptavidin phycoerythrin (Invitrogen) in 6× SSPE (0.9 M NaCl, 60 mM NaH_2_PO_4_, pH 7.4 and 6 mM Na_2_EDTA). Signal intensity detection was performed using the inbuilt CCD camera of the system and local backgrounds were subtracted by means of internal Geniom One software routines. Finally, signal intensities from probe sequences were used to determine methylation levels for each CpG site by calculating the CpG/(CpG+TpG) probe signal intensity ratio, representing the methylation level M/(M+U). Consequently, a fully methylated CpG should result in a signal intensity ratio of one whereas a fully unmethylated CpG should return a value of zero. However, owing to unspecific cross-hybridization mostly caused by low sequence complexity of sodium bisulfite treated sequences, not all probe sequences were suitable for further analysis. In order to identify probe sequences that could be used to accurately detect methylation levels from specific CpG sites, in-vitro methylated as well as unmethylated control pools were used for initial calibration. The two control pools were hybridized to individual sub-arrays followed by calculation of M/(M+U) probe signal intensity ratios for every represented CpG site. In the final microarray layout, probe sequence pairs were only included when the hybridization of the fully methylated control pool returned a probe signal intensity ratio above 0.75 and the hybridization of the fully unmethylated control pool returned a probe signal intensity ratio below 0.25.

### Clone sequencing

PCR products selected for validation were cloned into pCR4-TOPO via the TOPO TA cloning kit (Invitrogen) and the constructs were transformed into TOP10 cells. From each cloned PCR fragment, twelve different clones were picked and sequenced (GATC Biotech, Constance, Germany). Final methylation levels were determined from nine or more sequences obtained from each PCR fragment.

### Quantitative RT-PCR

Total RNA was isolated from each cell line using the RNeasy Mini Kit (Qiagen) according to the manufacturer's instructions. The one-step QuantiFast SYBR Green RT-PCR Kit (Qiagen) was used in combination with primers from the QuantiTect Primer Assay (Qiagen). Reactions were performed in 386 well format in triplicate with 25 ng total RNA per well in a LightCycler 480 (Roche). The endogenous controls ACTB1, GAPDH and TUBA3C were used for normalization.

### Array data deposition

The array data has been deposited with ArrayExpress (http://www.ebi.ac.uk/microarray-as/ae/) under the accession number E-MEXP-2698.

## Supporting Information

Table S1 Primer sequences for amplification of specified CGIs. Shown are ENSEMBL transcript and exon IDs used for CGI definition, as well as primer sequences used for PCR amplification of the specified CGIs.
(0.11 MB PDF)

Table S2 Methylation levels from all analyzed CpG sites. Methylation levels (M/[M+U])×100 are shown from all CpG sites of the investigated cell lines MCF-7_wt, MCF-7_ADR, OVCAR-5, OVCAR-4 and NCI/ADR-RES, together with the levels obtained from hybridization of 100% methylated, as well as 100% unmethylated, fragment pools. Columns SD show standard deviations.
(0.27 MB XLS)

Table S3 Gene expression levels relative to MCF-7_wt. Gene expression levels from indicated genes were determined from the cell lines MCF-7_wt, MCF-7_ADR, OVCAR-5, OVCAR-4 and NCI/ADR-RES. To allow comparison between cell lines, expression levels were normalized to a set of house-keeping genes within each cell line and are presented as fold-changes from MCF-7_wt. Columns SD show standard deviations from triplicates.
(0.12 MB PDF)

## References

[1] Minotti G, Menna P, Salvatorelli E, Cairo G, Gianni L (2004). Anthracyclines: molecular advances and pharmacologic developments in antitumor activity and cardiotoxicity.. Pharmacol Rev.

[2] Verma S, Dent S, Chow BJ, Rayson D, Safra T (2008). Metastatic breast cancer: the role of pegylated liposomal doxorubicin after conventional anthracyclines.. Cancer Treat Rev.

[3] Pisano C, Bruni GS, Facchini G, Marchetti C, Pignata S (2009). Treatment of recurrent epithelial ovarian cancer.. Ther Clin Risk Manag.

[4] Glasspool RM, Teodoridis JM, Brown R (2006). Epigenetics as a mechanism driving polygenic clinical drug resistance.. Br J Cancer.

[5] Bird A (2002). DNA methylation patterns and epigenetic memory.. Genes Dev.

[6] Saxonov S, Berg P, Brutlag DL (2006). A genome-wide analysis of CpG dinucleotides in the human genome distinguishes two distinct classes of promoters.. Proc Natl Acad Sci U S A.

[7] Jones PA, Baylin SB (2002). The fundamental role of epigenetic events in cancer.. Nat Rev Genet.

[8] Esteller M (2002). CpG island hypermethylation and tumor suppressor genes: a booming present, a brighter future.. Oncogene.

[9] Kantharidis P, El-Osta A, deSilva M, Wall DM, Hu XF (1997). Altered methylation of the human MDR1 promoter is associated with acquired multidrug resistance.. Clin Cancer Res.

[10] David GL, Yegnasubramanian S, Kumar A, Marchi VL, De Marzo AM (2004). MDR1 promoter hypermethylation in MCF-7 human breast cancer cells: changes in chromatin structure induced by treatment with 5-Aza-cytidine.. Cancer Biol Ther.

[11] Paige AJ, Brown R (2008). Pharmaco(epi)genomics in ovarian cancer.. Pharmacogenomics.

[12] Chekhun VF, Lukyanova NY, Kovalchuk O, Tryndyak VP, Pogribny IP (2007). Epigenetic profiling of multidrug-resistant human MCF-7 breast adenocarcinoma cells reveals novel hyper- and hypomethylated targets.. Mol Cancer Ther.

[13] Liscovitch M, Ravid D (2007). A case study in misidentification of cancer cell lines: MCF-7/AdrR cells (re-designated NCI/ADR-RES) are derived from OVCAR-8 human ovarian carcinoma cells.. Cancer Lett.

[14] Frommer M, McDonald LE, Millar DS, Collis CM, Watt F (1992). A genomic sequencing protocol that yields a positive display of 5-methylcytosine residues in individual DNA strands.. Proc Natl Acad Sci U S A.

[15] Mund C, Beier V, Bewerunge P, Dahms M, Lyko F (2005). Array-based analysis of genomic DNA methylation patterns of the tumour suppressor gene p16INK4A promoter in colon carcinoma cell lines.. Nucleic Acids Res.

[16] Calcagno AM, Fostel JM, To KK, Salcido CD, Martin SE (2008). Single-step doxorubicin-selected cancer cells overexpress the ABCG2 drug transporter through epigenetic changes.. Br J Cancer.

[17] Tubbs R, Barlow WE, Budd GT, Swain E, Porter P (2009). Outcome of patients with early-stage breast cancer treated with doxorubicin-based adjuvant chemotherapy as a function of HER2 and TOP2A status.. J Clin Oncol.

[18] Di Leo A, Biganzoli L, Claudino W, Licitra S, Pestrin M (2008). Topoisomerase II alpha as a marker predicting anthracyclines' activity in early breast cancer patients: ready for the primetime?. Eur J Cancer.

[19] Keith WN, Stallard S, Brown R (1990). Expression of mdr1 and gst-pi in human breast tumours: comparison to in vitro chemosensitivity.. Br J Cancer.

[20] Sharma D, Vertino PM (2004). Epigenetic regulation of MDR1 gene in breast cancer: CpG methylation status dominates the stable maintenance of a silent gene.. Cancer Biol Ther.

[21] Dejeux E, Ronneberg JA, Solvang H, Bukholm I, Geisler S (2010). DNA methylation profiling in doxorubicin treated primary locally advanced breast tumours identifies novel genes associated with survival and treatment response.. Mol Cancer.

[22] Chekhun VF, Kulik GI, Yurchenko OV, Tryndyak VP, Todor IN (2006). Role of DNA hypomethylation in the development of the resistance to doxorubicin in human MCF-7 breast adenocarcinoma cells.. Cancer Lett.

[23] Strathdee G, Davies BR, Vass JK, Siddiqui N, Brown R (2004). Cell type-specific methylation of an intronic CpG island controls expression of the MCJ gene.. Carcinogenesis.

[24] Strathdee G, Vass JK, Oien KA, Siddiqui N, Curto-Garcia J (2005). Demethylation of the MCJ gene in stage III/IV epithelial ovarian cancer and response to chemotherapy.. Gynecol Oncol.

[25] Tian K, Wang Y, Huang Y, Sun B, Li Y (2008). Methylation of WTH3, a possible drug resistant gene, inhibits p53 regulated expression.. BMC Cancer.

[26] Tian K, Jurukovski V, Wang XP, Kaplan MH, Xu H (2005). Epigenetic regulation of WTH3 in primary and cultured drug-resistant breast cancer cells.. Cancer Res.

[27] Tassone P, Tagliaferri P, Perricelli A, Blotta S, Quaresima B (2003). BRCA1 expression modulates chemosensitivity of BRCA1-defective HCC1937 human breast cancer cells.. Br J Cancer.

[28] Greenberg RA (2008). Recognition of DNA double strand breaks by the BRCA1 tumor suppressor network.. Chromosoma.

[29] Rathi A, Virmani AK, Schorge JO, Elias KJ, Maruyama R (2002). Methylation profiles of sporadic ovarian tumors and nonmalignant ovaries from high-risk women.. Clin Cancer Res.

[30] Muller HM, Widschwendter A, Fiegl H, Ivarsson L, Goebel G (2003). DNA methylation in serum of breast cancer patients: an independent prognostic marker.. Cancer Res.

[31] Muller HM, Fiegl H, Widschwendter A, Widschwendter M (2004). Prognostic DNA methylation marker in serum of cancer patients.. Ann N Y Acad Sci.

[32] Yuecheng Y, Hongmei L, Xiaoyan X (2006). Clinical evaluation of E-cadherin expression and its regulation mechanism in epithelial ovarian cancer.. Clin Exp Metastasis.

[33] Giacinti L, Claudio PP, Lopez M, Giordano A (2006). Epigenetic information and estrogen receptor alpha expression in breast cancer.. Oncologist.

[34] Jarvinen TA, Holli K, Kuukasjarvi T, Isola JJ (1998). Predictive value of topoisomerase IIalpha and other prognostic factors for epirubicin chemotherapy in advanced breast cancer.. Br J Cancer.

[35] Pakneshan P, Tetu B, Rabbani SA (2004). Demethylation of urokinase promoter as a prognostic marker in patients with breast carcinoma.. Clin Cancer Res.

[36] Chau BN, Diaz RL, Saunders MA, Cheng C, Chang AN (2009). Identification of SULF2 as a novel transcriptional target of p53 by use of integrated genomic analyses.. Cancer Res.

[37] Hampton OA, Den Hollander P, Miller CA, Delgado DA, Li J (2009). A sequence-level map of chromosomal breakpoints in the MCF-7 breast cancer cell line yields insights into the evolution of a cancer genome.. Genome Res.

[38] Ai L, Kim WJ, Demircan B, Dyer LM, Bray KJ (2008). The transglutaminase 2 gene (TGM2), a potential molecular marker for chemotherapeutic drug sensitivity, is epigenetically silenced in breast cancer.. Carcinogenesis.

[39] Hodges E, Smith AD, Kendall J, Xuan Z, Ravi K (2009). High definition profiling of mammalian DNA methylation by array capture and single molecule bisulfite sequencing.. Genome Res.

[40] Hatle KM, Neveu W, Dienz O, Rymarchyk S, Barrantes R (2007). Methylation-controlled J protein promotes c-Jun degradation to prevent ABCB1 transporter expression.. Mol Cell Biol.

[41] Jakoby WB (1978). The glutathione S-transferases: a group of multifunctional detoxification proteins.. Adv Enzymol Relat Areas Mol Biol.

[42] Masanek U, Stammler G, Volm M (1997). Messenger RNA expression of resistance proteins and related factors in human ovarian carcinoma cell lines resistant to doxorubicin, taxol and cisplatin.. Anticancer Drugs.

[43] Cheng X, Kigawa J, Minagawa Y, Kanamori Y, Itamochi H (1997). Glutathione S-transferase-pi expression and glutathione concentration in ovarian carcinoma before and after chemotherapy.. Cancer.

[44] Ikegami K, Ohgane J, Tanaka S, Yagi S, Shiota K (2009). Interplay between DNA methylation, histone modification and chromatin remodeling in stem cells and during development.. Int J Dev Biol.

[45] Fuks F (2005). DNA methylation and histone modifications: teaming up to silence genes.. Curr Opin Genet Dev.

[46] Mutskov V, Felsenfeld G (2004). Silencing of transgene transcription precedes methylation of promoter DNA and histone H3 lysine 9.. EMBO J.

[47] Li LC, Dahiya R (2002). MethPrimer: designing primers for methylation PCRs.. Bioinformatics.

[48] Baum M, Bielau S, Rittner N, Schmid K, Eggelbusch K (2003). Validation of a novel, fully integrated and flexible microarray benchtop facility for gene expression profiling.. Nucleic Acids Res.

