# Supplementary material for: High-Definition DNA Methylation Profiles from Breast and Ovarian Carcinoma Cell Lines with Differing Doxorubicin Resistance
Source: PLoS One. 2010 Jun 8;5(6):e11002. doi: 10.1371/journal.pone.0011002 (PMC2882327; doi:10.1371/journal.pone.0011002)
Supplement: Table S1 — Primer sequences for amplification of specified CGIs. Shown are ENSEMBL transcript and exon IDs used for CGI definition, as well as primer sequences used for PCR amplification of the specified CGIs. (0.11 MB PDF) [file pone.0011002.s001.pdf]

CpG Island	Ensembl transcript ID	Ensembl exon ID	5' end of forward primer relative to Exon (in bp)	Forward primer	Reverse primer	Annealing temp. (°C)	PCR fragement size (bp)
**ABCB1_A**	ENST00000265724	ENSE00001233532	94	TAGTTATTTGTGGTGAGGTTGATTG	ATCCCATAATAACTCCCAACTTTAC	60	265
**ABCB1_B**	ENST00000265724	ENSE00001233532	479	TTAGATTTAGGAGTTTTTGGAGTAG	AAAACAAAATTAAAAATCTAACAAC	60	218
**ABCG2**	ENST00000237612	ENSE00001342969	-216	GGGAGTGTTTGGTTTGTTTTT	TTCCTCCACAAACTACCTCCTTAC	60	548
**APAF1**	ENST00000333991	ENSE00001339839	-562	TTTTTATTAGGGGGAGTAGGA	AAACCCCACTACTAAACACAAAAAA	60	777
**APC**	ENST00000257430	ENSE00001913252	-205	AGGGTTAGGTAGGTTGTG	ACCAATACAACCACATATC	52	184
**ARHGEF2**	ENST00000361247	ENSE00001856287	-25	GGGGTTTTTTAAATTTGTTTTAGGA	AAAACAAAAAATCTAACTCCCCTC	60	200
**Aven**	ENST00000306730	ENSE00001235829	665	GGGTTAGGAATTTAGAGGTTAAGGT	ACACAAACAATTAACAAAACAAACC	60	198
**BAD**	ENST00000309032	ENSE00001518739	-618	TTTAGTTAGGGAGGAGGTTTTTTTT	CTTCAACCCCTCTAAACCTTAATTT	60	321
**BIRC5**	ENST00000301633	ENSE00001305879	-238	TGGTTTTGAATTTTAGGATTTAAGTG	AAAAAACTACCAAACAAAAAACAAC	60	398
**BRCA1**	ENST00000357654	ENSE00001871077	-180	AGATTGGGTGGTTAATTTAGAGTTT	ATAATATCCCCCTCAAAACATATTC	60	546
**CDH1**	ENST00000261769	ENSE00001859184	-128	ATTTAGATTTTAGTAATTTTAGGTTAGAGG	CAAACTCACAAATACTTTACAATTCC	60	221
**CDKN2A**	ENST00000304494	ENSE00001833804	-80	GTGGGGAGGAGTTTAGTTTTTTT	TACAAACCCTCTACCCACCTAAATC	60	519
**DNAJC15_A**	ENST00000379221	ENSE00001812089	-321	TTTTTGAGTAGTTGGGATTATAGGG	CTCCACCCCCAAATAAATAACTATAT	60	216
**DNAJC15_B**	ENST00000379221	ENSE00001812089	120	GTTTGGGGAGGGATTAGG	CTAACAAAACTCACCAATCTCTAC	60	426
**ESR1_A**	ENST00000206249	ENSE00001877305	-313	AGTGTAGTTTTTTTTAGGGTTATTTTATGT	AACCTCCAACTTTAAATACTAATCTCC	60	432
**ESR1_B**	ENST00000206249	ENSE00001877305	34	AGGGTAAGGTAATAGTTTTTGGT	AACTTACTACTATCCAAATACACCTC	60	474
**FANCF**	ENST00000327470	ENSE00001308799	33	GGAATTTTTTTTGTAGTATTTGGAT	AAACTCTCTTAAAATATCTCCTCATC	60	396
**FOXO3A**	ENST00000343882	ENSE00001212218	-494	AGGTTAGGGATTTTTTGGTATTAGG	CAACAACACAAAATTATAAACACAC	60	471
**GSTP1**	ENST00000398606	ENSE00001533853	2	GGAAAGAGGGAAAGGTTTTTT	CCATACTAAAAACTCTAAACCCCATC	60	302
**HIC1**	ENST00000322941	ENSE00001599905	-624	GGGTAGGGGAGTTTAGGGTT	ATTACCCCAATTAAAAAAAATAATAC	52	553
**IGFBP3**	ENST00000275521	ENSE00001888357	-322	GTGTTGAGTTGGTTAGGAGTGATT	AAACTATAAAATCCAAACAAAAAAC	60	386
**MLH1**	ENST00000231790	ENSE00001943203	-541	TTTTTTTTAGGAGTGAAGGAGGTTA	TCCAACCAATAAAAACAAAAATACC	60	443
**MSH2**	ENST00000233146	ENSE00001828808	-84	GTTTTGTAGTTGAGTAAATATAGAAAGGAGTT	AACCTCCTCACCTCCTAATTAAAAA	60	302
**PLAU_A**	ENST00000372764	ENSE00001820360	-261	GGTTAGGTGTATGGGAGGAAGTA	ATAACCAAACTCCCCAACTATCTCT	60	541
**PLAU_B**	ENST00000372764	ENSE00001820360	255	AGAGATAGTTGGGGAGTTTGGTTAT	TCCTTAAACAACATCAATCAAAACA	60	239
**PTEN**	ENST00000371953	ENSE00001456562	224	TTATATTGGGTATGTTTAGTAGAGTTTG	AACTTCCATCATAACTACAACTTCC	60	326
**RAB6C**	ENST00000410061	ENSE00001588213	-83	TATTATTGTTTTTAGTATATATGTTTTGTG	AAACACCAACTTAAATTTCCTCAAC	60	589
**RALBP1**	ENST00000019317	ENSE00001496923	-2	TTAGTTTGGTTTAATTGGTTGGAA	CAAACTACAACACCCAAAAAACAC	60	323
**RASSF1**	ENST00000359365	ENSE00001889507	-123	GGAGGGAAGGAAGGGTAAGG	CAACTCAATAAACTCAAACTCCC	60	260
**SULF2**	ENST00000359930	ENSE00001828934	-202	TGTAGTTGTTGGTGAGTTTTTTGTT	CAACCACACACATTTACCATTAAAA	60	326
**TGM2**	ENST00000361475	ENSE00001922845	-124	TTGTATTTTGGGTTAGTTGTGTGTT	CTCTCCAAATCAAAACTTAAAAATTC	60	406
**TP73_A**	ENST00000378295	ENSE00001910421	-1009	GGTTTTTTAGTTAGGGTTTGGTGTA	AAAACTTACCACCCACTTCTCCTATA	60	403
**TP73_B**	ENST00000378295	ENSE00001910421	-695	GGGTTGGGAGAGTAGTTTTTAGAG	AATCCAAAAAACAAAAAATATAACC	60	203
