# Supplementary material for: High-Definition DNA Methylation Profiles from Breast and Ovarian Carcinoma Cell Lines with Differing Doxorubicin Resistance
Source: PLoS One. 2010 Jun 8;5(6):e11002. doi: 10.1371/journal.pone.0011002 (PMC2882327; doi:10.1371/journal.pone.0011002)
Supplement: Table S3 — Gene expression levels relative to MCF-7_wt. Gene expression levels from indicated genes were determined from the cell lines MCF-7_wt, MCF-7_ADR, OVCAR-5, OVCAR-4 and NCI/ADR-RES. To allow comparison between cell lines, expression levels were normalized to a set of house-keeping genes within each cell line and are presented as fold-changes from MCF-7_wt. Columns SD show standard deviations from triplicates. (0.12 MB PDF) [file pone.0011002.s003.pdf]

Gene name	MCF-7_wt	MCF-7_ADR	*SD*	OVCAR-5	*SD*	OVCAR-4	*SD*	NCI/ADR-RES	*SD*
ABCB1	1.0E+00	4.1E+03	*2.0E+03*	2.5E-01	*8.0E-02*	1.8E-01	*1.0E-01*	2.9E+04	*7.0E+03*
GSTP1	1.0E+00	1.8E+03	*6.0E+02*	4.2E+03	*7.0E+02*	6.6E+03	*3.0E+03*	1.8E+03	*4.0E+02*
PLAU	1.0E+00	4.8E+01	*2.0E+01*	3.6E+02	*2.0E+02*	4.4E+01	*2.0E+01*	5.8E+01	*2.0E+01*
TGM2	1.0E+00	1.4E+01	*3.0E+00*	6.5E+01	*1.0E+01*	4.5E+00	*1.0E+00*	1.7E+01	*3.0E+00*
IGFBP3	1.0E+00	8.5E+00	*3.0E+00*	3.7E-01	*6.0E-02*	6.4E+01	*1.0E+01*	1.9E+01	*3.0E+00*
RASSF1	1.0E+00	1.2E+00	*4.0E-01*	3.3E+00	*3.0E-01*	1.0E+00	*3.0E-01*	1.8E+00	*3.0E-01*
HIC1	1.0E+00	1.1E+00	*4.0E-01*	2.0E+00	*2.0E-01*	9.7E-01	*3.0E-01*	1.5E+00	*2.0E-01*
APC	1.0E+00	7.4E-01	*2.0E-01*	9.8E-01	*2.0E-01*	1.3E+00	*3.0E-01*	6.9E-01	*2.0E-01*
TOP2A	1.0E+00	6.9E-01	*3.0E-01*	2.0E+00	*5.0E-01*	6.9E-01	*2.0E-01*	5.1E-01	*1.0E-01*
TP73	1.0E+00	5.6E-01	*3.0E-01*	2.0E+00	*6.0E-01*	1.2E+00	*4.0E-01*	6.4E-01	*2.0E-01*
RAB6C	1.0E+00	4.6E-01	*2.0E-01*	1.5E+00	*1.0E-01*	1.1E+00	*3.0E-01*	5.6E-01	*2.0E-01*
BRCA1	1.0E+00	3.2E-01	*1.0E-01*	9.1E-01	*2.0E-01*	4.3E-01	*2.0E-01*	3.3E-01	*5.0E-02*
ABCG2	1.0E+00	1.3E-01	*3.0E-02*	5.6E+00	*1.0E+00*	1.0E-01	*2.0E-02*	1.6E-01	*3.0E-02*
ESR1	1.0E+00	2.3E-03	*1.0E-03*	3.5E-02	*7.0E-03*	1.4E-02	*5.0E-03*	9.1E-03	*2.0E-03*
DNAJC15	1.0E+00	1.4E-03	*9.0E-04*	1.0E+00	*6.0E-01*	8.8E-02	*4.0E-02*	2.7E-03	*2.0E-03*
SULF2	1.0E+00	1.4E-04	*3.0E-05*	1.2E-01	*2.0E-02*	4.3E-02	*2.0E-02*	2.1E-04	*9.0E-05*
CDH1	1.0E+00	1.4E-04	*6.0E-05*	9.6E-01	*8.0E-02*	1.7E-01	*4.0E-02*	4.6E-04	*6.0E-05*
